# A74 URGENCY FOR BOWEL MOVEMENTS IS A HIGHLY DISCRIMINATORY SYMPTOM FOR ACTIVE DISEASE IN PERSONS WITH IBD (THE MANITOBA LIVING WITH IBD STUDY)

**DOI:** 10.1093/jcag/gwac036.074

**Published:** 2023-03-07

**Authors:** A Kulyk, L A Shafer, L A Graff, J Stone, K Witges, L E Targownik, C Bernstein

**Affiliations:** 1 Internal Medicine, University of Manitoba; 2 University of Manitoba IBD Clinical and Research Centre; 3 Department of Clinical Health Psychology, University of Manitoba; 4 Internal Medicine, University of Toronto, Winnipeg, Canada

## Abstract

**Background:**

The Inflammatory Bowel Disease Symptom Inventory (IBDSI) is a validated patient self-reported measure used to assess IBD disease activity.

**Purpose:**

We aimed to assess the prevalence of symptoms, and examine which symptoms are most associated with disease activity as measured by a symptom index and objective measure of inflammation.

**Method:**

The Manitoba Living with IBD Study is a prospective study of 156 participants with confirmed IBD who completed bi-weekly Inflammatory Bowel Disease Symptom Inventory (IBDSI) surveys. Relative risks (RR) (with 95% confidence interval (CI)), positive and negative predictive values (PPV, NPV), and area under receiver operator curve (AUC) were reported for each symptom to predict active disease defined as: (1) active IBDSI, (2) self-reported flare, and (3) elevated fecal calprotectin (FCAL) (>250µg/g). Analyses were undertaken following stratification based on sex, and disease type (Crohn’s disease (CD) and ulcerative colitis (UC)).

**Result(s):**

In total, 69.2% of participants were female; 64.7% had CD. Fatigue was the most prevalent symptom in both inactive and active disease, across all 3 disease measures (IBDSI: 24.5% and 75.1%, self-reported flare: 42.2% and 72.2%, FCAL: 46.0% and 60.6%). Absence of fatigue had a high NPV for active IBDSI and self-reporting a flare in both CD and UC. Urgency had a consistently strong NPV, RR and AUC across all three disease measures in both IBD subtypes and sexes. The number of loose/liquid bowel movements predicted elevated FCAL in UC (RR in men = 3.5, 95% CI 1.2-9.9, RR in women = 1.8, 95% CI 1.2-2.7, AUC in men = 0.73, 95% CI 0.59-0.84, AUC in women = 0.65, 95% CI 0.56-0.72), as did blood in stool in females with UC (RR = 1.8, 95% CI 1.2-2.7). In men with CD, excessive bowel gas (RR = 2.0, 95% CI 1.2-3.4) and urgency (RR = 3.9, 95% CI 1.6-9.3) best predicted an elevated FCAL. No symptom was strongly predictive of elevated FCAL in females with CD.

**Conclusion(s):**

Fatigue was the most prevalent symptom irrespective of disease activity. Urgency stood out as being consistently associated with disease activity, irrespective of the disease measure. Individual symptoms have different impacts on subjective (IBDSI) and objective (FCAL) measures in IBD.

**Please acknowledge all funding agencies by checking the applicable boxes below:**

None

**Disclosure of Interest:**

None Declared

